# Intra-aortic balloon pump: is the tide turning?

**DOI:** 10.1186/s13054-018-2266-8

**Published:** 2018-12-18

**Authors:** Sandro Gelsomino, Daniel M. Johnson, Roberto Lorusso

**Affiliations:** 1 Department of Cardiothoracic Surgery, Maastricht University Hospital, Maastricht, The Netherlands; 2 Department of Cardiothoracic Surgery, Cardiovascular Research Institute Maastricht—CARIM, Universiteitssingel 50, 6229 ER Maastricht, The Netherlands; 3 Institute of Cardiovascular Sciences, University of Birmingham, Birmingham, UK

Since the intra-aortic balloon pump (IABP) was used for the first time by Kantrowitz et al. [1] there has been controversy regarding its beneficial effects. In fact, even a report where Kantrowitz himself is senior author states [2]:

… Precise indications for initiation and termination of balloon counterpulsation remain in doubt.

However, after years of honest service, the IABP has been struck by a scientific thunderstorm called “SHOCK II”, which has seriously questioned the use of this assist device in cardiogenic shock complicating acute myocardial infarction [3]. After the initial “SHOCK” of this trial there are still a number of questions remaining regarding the utility of the IABP, as well as a number of different “camps”. There are both the “storm riders”, who have always believed that the IABP had limited use, and, on the other side, the “honest IABP believers” who claim that successful use of IABP counterpulsation has been life-saving in many patients.

The majority of physicians, however, are in the middle, finding themselves “between a rock and hard place”. These physicians are overwhelmed by the fear of not adhering to guidelines more than being really convinced of the lack of benefit of IABP use [4, 5]. The net result of this “hurricane” is that, in clinical practice in Europe and the United States, the utilization rate of the IABP has been decreasing over the last few years (Fig. 1).

**Fig. 1:**
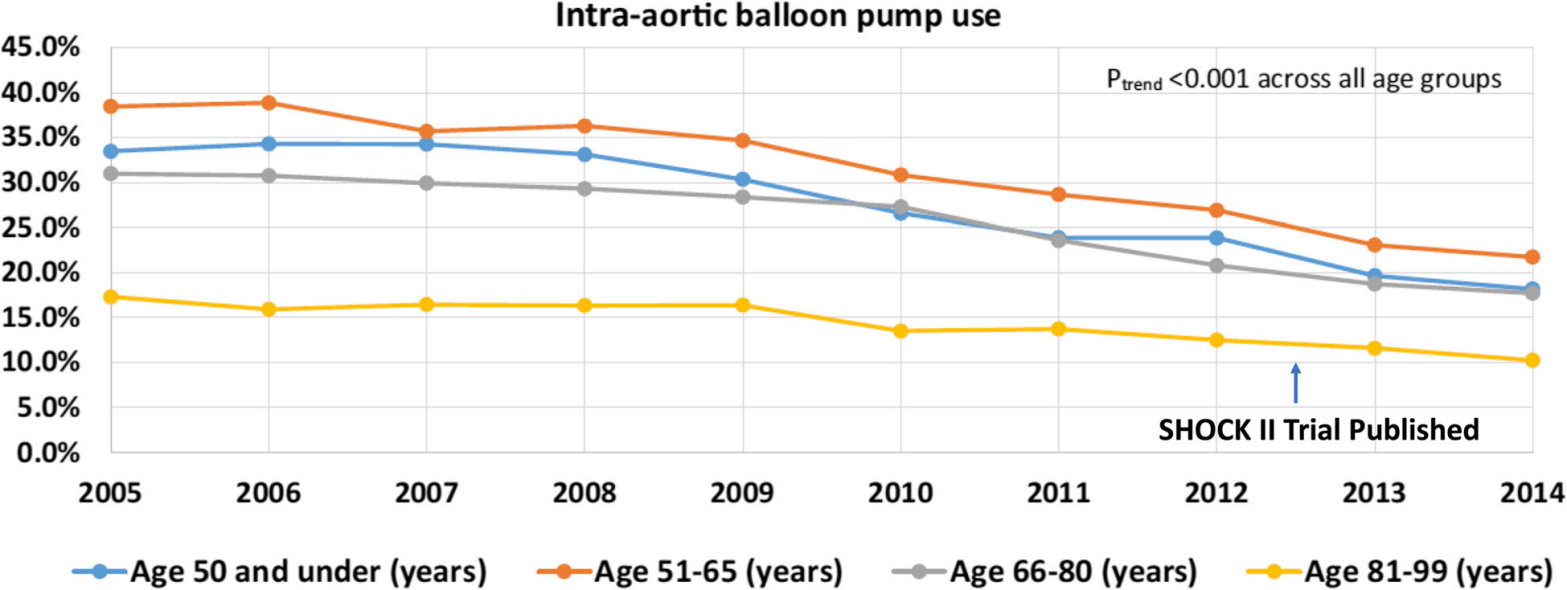
Trends in IABP use in the United States. Data extracted from cost and utilization project (HCUP) database from National Institutes of Health, which collects data from all over the United States. Hospitalizations to identify admissions for cardiogenic shock between 2005 and 2014. Adapted from [14] with permission

Remaining neutral between the opposing views and recognizing the unquestionable scientific value of the SHOCK II trial, doubt has been raised on whether our clinical convictions can be straightforwardly driven by evidence coming just from a single randomized trial.

A number of recent studies have shown that there is some sun on the horizon regarding use of the IABP. A recent meta-analysis, for example, included 9212 patients and investigated the utility of the IABP when implanted preoperatively in patients undergoing coronary bypass graft surgery [6]. The results of this analysis strongly indicate that there is benefit in using the IABP under these conditions, with the relative risk reduction of mortality being more than 4%. Furthermore, the risk of MI and renal failure were reduced when IABP treatment was instigated and both intensive care and total hospital stays were reduced, also indicating a possible economic benefit, as well as health benefit, of using the IABP [6].

Likewise, a recent study by Yang et al. [7], carried out in 416 patients with LV dysfunction undergoing off-pump coronary bypass grafting, showed that a preoperative IABP was linked with a lower 30-day mortality.

Interestingly, Iqbal et al. [8] recently carried out an observational analysis of 174 patients (with 55 patients receiving IABP) successfully resuscitated following an out-of-hospital cardiac arrest. In this study, the use of IABP therapy in the postresuscitation period was associated with improved functional recovery and outcomes, although the mortality rate was not different between the IABP and non-IABP groups [8].

Imamura et al. [9] recently showed that an elevation in central venous pressure and a lower heart rate were a predictor for significant hemodynamic response to IABP treatment in a population of decompensated heart failure patients. A very recent study [10] indicated that the IABP was associated with a lower risk of 30-day mortality in patients with acute myocardial infarction complicated by cardiogenic shock, in whom percutaneous coronary intervention was unsuccessful, whilst a higher risk of death was seen in patients where PCI had been successful. Taken together, these data indicate that improved patient selection may greatly influence outcomes.

Interestingly, use of the IABP together with other support systems, such as extracorporeal membrane oxygenation (ECMO), has also been receiving increased attention over recent years [11]. For example, a recent study by Meani et al. [12] showed the potential utility of the IABP to reverse aortic valve closure and impaired left ventricular unloading that occurs during V-A ECMO support, whilst Bréchot et al. [13] showed that the association of IABP with V-A ECMO was associated with a lower frequency of pulmonary edema. Further research, both at the basic and the clinical level, is, however, required to fully understand the utility of such combination therapy.

Is the tide turning? At this stage, it is too early to say and we should be prudent, whilst at the same time critical, when examining studies. Nevertheless, the heavy debate on appropriate use of the IABP needs new lifeblood from numerous avenues including cardiologists, intensivists, anesthesiologists and cardiac surgeons. These specialties need to work together to actively contribute to a rigorous and objective data collection/examination/analysis. Furthermore, a key role needs to be played by companies involved in IABP development, who should, in our opinion, show an interest in gaining new scientific evidence to aid the scientific community in filling the considerable gap currently existing between guidelines and clinical practice.

In conclusion, maybe the time is right for new well-designed clinical trials to cause an “After-SHOCK II” in the field of IABP support. Only these data will properly inform the community whether there is some nice weather on the horizon or whether we just have a temporary rainbow.

## Abbreviation

IABP: Intra-aortic balloon pump
